# Risk factors and prediction model for nonalcoholic fatty liver disease in northwest China

**DOI:** 10.1038/s41598-022-17511-6

**Published:** 2022-08-16

**Authors:** Danting Li, Meiyu Zhang, Shengli Wu, Huiwen Tan, Nong Li

**Affiliations:** 1 Department of Health Management Center, West China Hospital, Sichuan University, Chengdu, 610041 Sichuan China; 2 Department of Health Management Center, People’s Hospital of Karamay, Karamay, 834000 Xinjiang China; 3 Department of Endocrinology and Metabolism, People’s Hospital of Karamay, Karamay, 834000 Xinjiang China; 4 Department of Endocrinology and Metabolism, West China Hospital, Sichuan University, Chengdu, 610041 Sichuan China

**Keywords:** Health care, Risk factors

## Abstract

In recent years, nonalcoholic fatty liver disease (NAFLD) has become the most important chronic liver disease worldwide. The prevalence of NAFLD in China has also increased year by year. This study aimed to detect NAFLD early by developing a nomogram model in Chinese individuals. A total of 8861 subjects who underwent physical examination in Karamay and were 18 to 62 years old were enrolled. Clinical information, laboratory results and ultrasound findings were retrieved. The participants were randomly assigned to the development set (n = 6203) and the validation set (n = 2658). Significant variables independently associated with NAFLD were identified by least absolute shrinkage and selection operator (LASSO) regression and the multiple logistic regression model. Six variables were selected to construct the nomogram: age, sex, waist circumference (WC), body mass index (BMI), alanine aminotransferase (ALT), triglycerides and glucose index (TyG). The area under the receiver operating characteristic curve (AUROC) of the development set and validation set was 0.886 and 0.894, respectively. The calibration curves showed excellent accuracy of the nomogram model. This physical examination and laboratory test-based nomogram can predict the risk of NAFLD intuitively and individually.

## Introduction

Nonalcoholic fatty liver disease (NAFLD) is a multisystemic disease associated with diverse metabolic comorbidities. The entire disease spectrum ranges from hepatic steatosis and nonalcoholic steatohepatitis (NASH) to advanced fibrosis and cirrhosis, finally may lead to hepatocellular carcinoma^1,2^. At present, NAFLD has become the leading cause of chronic liver disease in China, which not only seriously endangers public health, but also brings a huge burden to the health care system^3^.

Statistically^4^, the overall global prevalence of NAFLD diagnosed by imaging was estimated to be 25.24%. The highest prevalence of NAFLD was reported in the Middle East (31.79%), while the lowest rate was reported in Africa (13.48%), and the prevalence rate in Asia was above the average (27.37%). According to a recent meta-analysis of data from 1999 to 2019^5^, the overall prevalence of NAFLD in adults in Asia is now estimated to be 29.62%, regardless of the diagnostic method. In China, the prevalence rate has been estimated to be 29.81%, and the prevalence of NAFLD has been increasing over the years, which has surpassed that in the Western world^4^. Based on a comprehensive search of the literature from 1999 to 2018, Zhou et al. revealed a rapid growth in the NAFLD population in China. Figures showed that the prevalence of NAFLD in China in the early 2000s was approximately 23.8%; nevertheless, the prevalence rate reached 32.9% in 2018^6^. The reported prevalence of NAFLD was heterogeneous in different regions of China, which is not only affected by the diagnostic methods but also influenced by the discrepancy in economic condition and lifestyle.

The diagnosis of NAFLD requires evidence of hepatic steatosis by either imaging or histology and the absence of other causes of hepatic fat accumulation, such as alcohol abuse, hepatitis B and C, and medication use^7^. The diagnosis and staging of NAFLD rely heavily on liver biopsy, especially for the diagnosis of NASH^8^. However, liver biopsy is expensive and invasive and is not feasible as a general diagnostic procedure^7^. Therefore, imaging techniques are still the main diagnostic methods, such as ultrasound, controlled attenuation parameter (CAP) and vibration controlled transient elastography (VCTE)based on Fibro Scan, X-ray computed tomography (CT), magnetic resonance imaging (MRI), magnetic resonance spectroscopy (MRS), etc.^9–11^. Various imaging modalities are available, but liver ultrasound is a pragmatic and widely accepted first-line investigation.

Nevertheless, the sensitivity and specificity of different diagnostic methods vary, and due to economic considerations, not all methods can be widely used. For example, MRS is highly accurate for even minimal amounts of steatosis, but its widespread application is hampered by its cost and availability^12^. Because of the high prevalence and potentially silent progression of NAFLD, the early identification and management of patients at risk are important. Clinical prediction models based on data mining are helpful to improve the diagnosis and monitoring of diseases. These models facilitate the detection of high-incidence diseases and can be conveniently installed on the computers of medical institutions for clinical use, which is economic and practical and can be applied extensively^13^.

A nomogram is a graphical presentation format for disease prediction models using different clinical data, which has been widely applied in the risk prediction of various diseases and provides accurate individualized estimates of outcomes^14^. However, the application of nomograms in NAFLD is still rare. Furthermore, considering the different prevalence of NAFLD in different regions, the clinical data included in the nomogram should also be different.

The purpose of the present study was to assess the risk factors for NAFLD and to develop a clinical prediction model based on clinical and laboratory data that used a nomogram as a presentation to detect NAFLD in the general population in Karamay, Xinjiang.

## Materials and methods

### Subjects

This cross-sectional study was conducted among adults who received annual health examinations at the Health Management Center of Xinjiang People’s Hospital of Karamay from January 2018 to March 2019. A total of 8861 subjects with complete hepatic ultrasonography examination data were included in the study. There were 5390 males and 3471 females aged 18–62 years, with a median of 38 (31–47) years, including 3261 subjects with NAFLD and 5600 subjects without NAFLD. NAFLD was diagnosed by ultrasonographic findings. Exclusion criteria included (1) significant alcohol intake (> 140 g/week for men and 70 g/week for women); (2) hepatitis B and C by serologic and virologic criteria; (3) drug-induced liver disease; (4) autoimmune liver disease; and (5) metabolic liver disorders such as Wilson’s disease^7^.

The study followed the principles expressed in the World Medical Association Declaration of Helsinki and the International Ethical Guidelines for Biomedical Research Involving Subjects (GIOMS, Geneva, 1993) and Chinese clinical research management regulations. The study program was approved by the medical ethics committee of People’s Hospital of Karamay (No. JK2019-1). Informed consent was obtained from all subjects.

### Methods

#### Clinical information

Information about sex, age, ethnicity, height, weight, blood pressure, waist circumference (WC), history of alcohol consumption and previous medical history was collected. The subjects took off shoes on an empty stomach and wore light clothes to measure their height and weight, and the readings were accurate to 0.5 cm and 0.5 kg respectively. Body mass index (BMI) was calculated as weight (kg) divided by height squared (m^2^). Waist circumference was measured using plastic tape at the midpoint between the lowest rib and the superior border of the iliac crest as the subject exhaled normally. BMI ≥ 25.0 kg/m^2^ was divided into the obesity group. Abdominal obesity was grouped according to WC: females > 80 cm and males > 90 cm were the obesity1 group^15^.

#### Laboratory tests

Venous blood samples were collected after 12 h overnight fast, and fasting plasma glucose (FPG), alanine aminotransferase (ALT), aspartate aminotransferase (AST), albumin (ALB), triglycerides (TGs), total cholesterol (TC), creatinine (Cre), serum potassium (K), and serum sodium (Na) were measured using standard techniques. The triglyceride glucose (TyG) index was calculated as ln (fasting TG (mg/dL) × FPG (mg/dL)/2)^16^.

#### Ultrasound examination and diagnosis of NAFLD

All subjects underwent abdominal ultrasonography to evaluate for fatty liver by trained sonographers who were blinded to the clinical data using a GE LOGIQ E9 apparatus equipped with a convex 1.0–5.0 MHz probe. Diffuse fatty liver can be defined as enhanced near-field echo (“bright liver”), attenuated far-field echo, increased liver and kidney echo contrast, intrahepatic vessel blurring and deep attenuation. NAFLD was diagnosed after the exclusion of diffuse fatty liver caused by alcohol, virus, autoimmunity, drugs and other factors^16^.

### Statistical analysis

First, data preprocessing is carried out. The variables irrelevant to this study or variables with a number of missing values were deleted (missing proportion more than 1.5%). The mode was used to fill the null values in categorical variables, and the mean value was used to fill the null values of continuous variables.

Clinical observation data were collected in an Excel database. The statistical analysis was performed with SPSS software (version 26.0) and R software (version 3.6.3). Continuous variables were expressed as the mean ± standard deviation for normally distributed variables or as the median ± interquartile range for nonnormally distributed variables. The *t*-test or Mann–Whitney* U* test was used for comparisons between the two groups. Moreover, categorical variables were presented using frequencies or percentages and were assessed by the chi-square test. The subjects were randomly assigned to the development group and the validation group at a ratio of 7:3. Least absolute shrinkage and selection operator (LASSO) regression was used to screen the influencing factors related to NAFLD, and the independent predictors were further determined by multivariate logistic regression analysis. The RMS package was downloaded to build the nomogram with predictive variables. The diagnostic accuracy of the nomogram prediction model was evaluated in terms of the area under the receiver operating characteristic curve (AUROC) and Hosmer–Lemeshow goodness of fit test. All *p* values presented were two-tailed, and differences were considered statistically significant at *p* < 0.05.

## Results

### The missing values of variables

**Figure 1:**
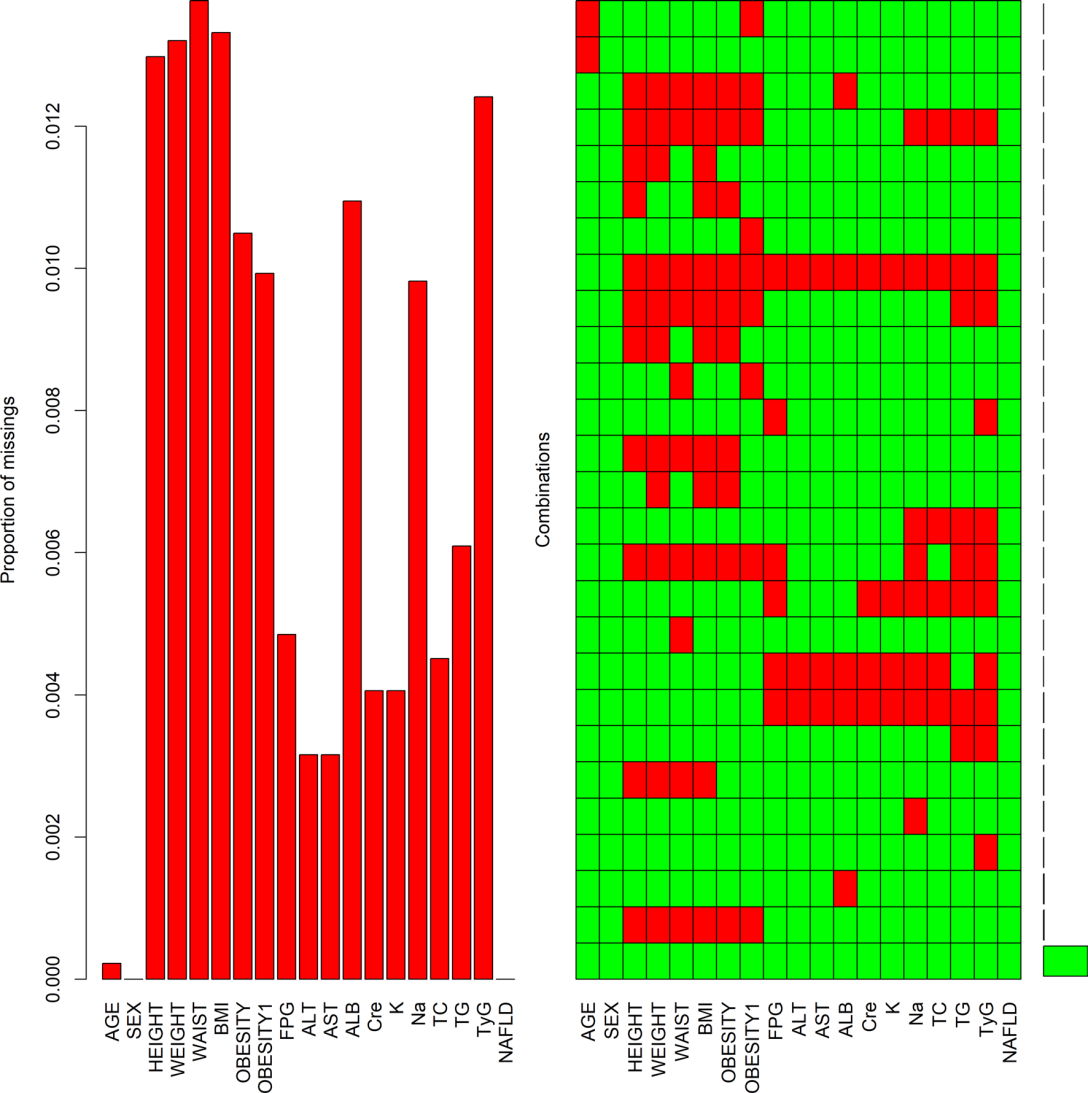
Missing values of variables. *BMI* body mass index, *FPG* fasting plasma glucose, *ALT* alanine aminotransferase, *AST* aspartate aminotransferase, *ALB* albumin, *Cre* creatinine, *TC* total cholesterol, *TG* triglycerides, *TyG* triglycerides and glucose index, *NAFLD* nonalcoholic fatty liver disease. Obesity: BMI ≥ 25.0 kg/m^2^ ; obesity1: WC > 80 cm for female or WC > 90 cm for male.

A total of 1364 missing values were detected by a missing value test on the collected NAFLD data. Figure 1 shows the missing data of 18 variables. The bar chart displays the missing proportion of all variables, showing that the missing percentages were all below 1.5%. The graph was sorted by samples missing components, and the red represents missing values, revealing that the sample with no missing values had the highest proportion.

### Characteristics of the study cohort (Table 1)

**Table 1:** Characteristics of the development set and validation set.

Characteristics	Development set (n = 6203)	Validation set (n = 2658)	*p*-value
Age (years)	39.1 ± 9.2	39.0 ± 9.2	0.734
**Sex, n (%)**			
Male	3758 (60.6)	1634 (61.5)	0.445
Female	2445 (39.4)	1024 (38.5)	
Height (cm)	168.7 ± 8.5	168.8 ± 8.6	0.629
Weight (kg)	71.56 ± 14.59	71.71 ± 14.87	0.667
Waist (cm)	86.89 ± 12.04	87.09 ± 12.24	0.485
BMI (kg/m^2^)	25.01 ± 4.07	25.01 ± 4.03	0.981
**Obesity, n (%)**			
No	3313 (53.4)	1386 (52.1)	0.284
Yes	2890 (46.6)	1272 (47.9)	
**Obesity1, n (%)**			
No	3240 (52.2)	1402 (52.7)	0.674
Yes	2963 (47.8)	1256 (47.3)	
FPG (mmol/L)	5.68 ± 1.53	5.75 ± 1.70	0.068
ALT (U/L)	27.90 ± 23.49	27.87 ± 22.80	0.957
AST (U/L)	21.79 ± 12.75	21.56 ± 11.02	0.411
ALB (g/L)	41.45 ± 15.01	41.72 ± 14.63	0.437
Cre (μmol/L)	74.45 ± 14.85	74.67 ± 14.27	0.526
K (mmol/L)	4.79 ± 1.36	4.80 ± 1.28	0.742
Na (mmol/L)	140.57 ± 7.92	140.77 ± 6.50	0.252
TC (mmol/L)	4.73 ± 1.03	4.74 ± 0.96	0.740
TG (mmol/L)	1.79 ± 1.32	1.79 ± 1.32	0.878
TyG	7.19 ± 0.69	7.19 ± 0.71	0.630
**NAFLD, n (%)**			
No	3916 (63.1)	1684 (63.4)	0.859
Yes	2287 (36.9)	974 (36.6)	

Obesity: BMI ≥ 25.0 kg/m^2^; obesity1: WC > 80 cm for female or WC > 90 cm for male.

*BMI* body mass index, *FPG* fasting plasma glucose, *ALT* alanine aminotransferase, *AST* aspartate aminotransferase, *ALB* albumin, *Cre* creatinine, *TC* total cholesterol, *TG* triglycerides, *TyG* triglycerides and glucose index, *NAFLD* nonalcoholic fatty liver disease.

The 8861 subjects were randomly divided into the development set (n = 6203) and the validation set (n = 2658) at a ratio of 7:3. There were no statistically significant differences in clinical information or laboratory results between the development set and the validation set (*P* > 0.05).

### Independent predictors in the development set

**Figure 2:**
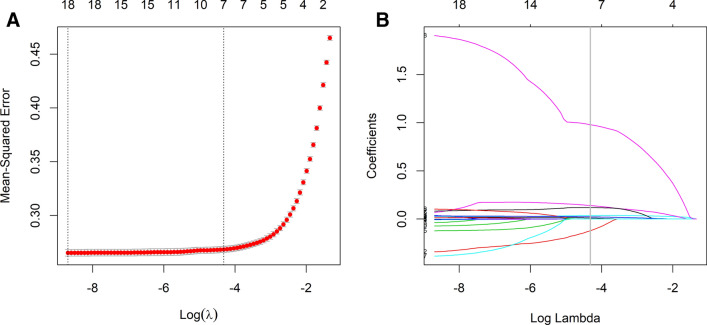
Variable selection by the LASSO binary logistic regression model. (**A**) Optimization parameters (lambda) of the LASSO model were selected using tenfold cross-validation. The mean-squared error was plotted versus log (lambda). (**B**) LASSO coefficient profiles of the 18 variables.

Considering the collinearity among some of the included variables, LASSO regression analysis was applied to screen out the predictive variables from those shown in Table 1, and seven variables with nonzero coefficients were obtained, as shown in Fig. 2. These seven variables were subjected to multivariate logistic regression analysis to identify independent factors strongly associated with NAFLD. The results showed that there were six variables in the development set that could be used as independent predictors of NAFLD (Table 2), including age, sex, waist circumference, BMI, ALT and TyG. Among them, female sex was a protective factor, and the other variables were risk factors.

**Table 2:** Multivariate logistic regression analysis for risk factors of NAFLD.

Variables	β	SE	Wald	df	*p*-value	OR	95% CI
Age (years)	0.022	0.004	5.374	1	< 0.001	1.022	1.014–1.030
Sex (female)	-0.269	0.094	-2.872	1	0.004	0.764	0.636–0.918
Waist (cm)	0.033	0.007	4.945	1	< 0.001	1.033	1.020–1.047
BMI (kg/m^2^)	0.191	0.018	10.688	1	< 0.001	1.210	1.169–1.254
ALT (U/L)	0.029	0.002	13.506	1	< 0.001	1.029	1.025–1.034
TyG	1.057	0.064	16.431	1	< 0.001	2.877	2.538–3.266

*BMI* body mass index, *ALT* alanine aminotransferase, *TyG* triglycerides and glucose index, *OR* odds ratio, *NAFLD* nonalcoholic fatty liver disease.

### Establishment of the nomogram

**Figure 3:**
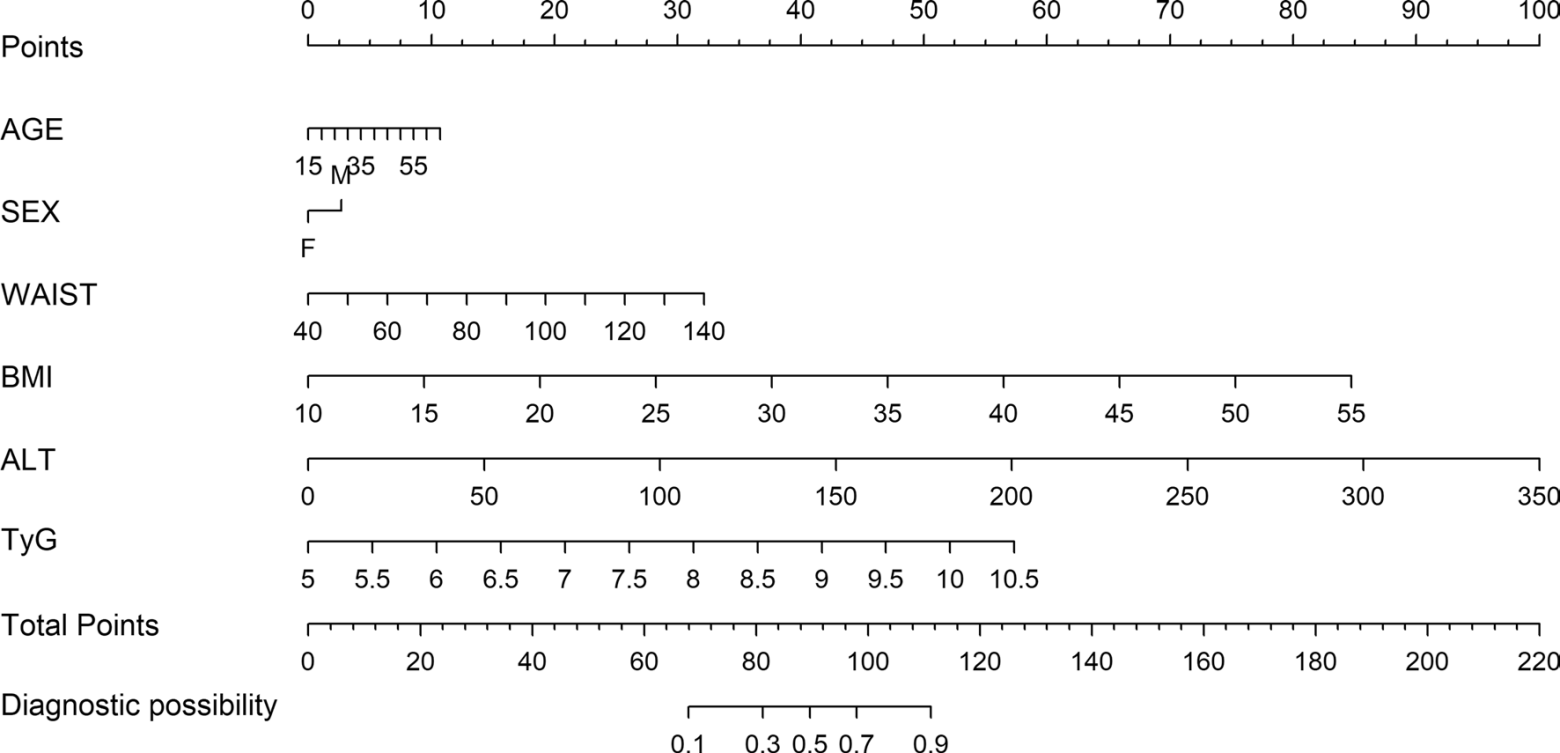
Nomogram to predict the risk of NAFLD. BMI, body mass index; ALT, alanine aminotransferase; TyG, triglycerides and glucose index; NAFLD, nonalcoholic fatty liver disease.

Based on the results of multivariate logistic regression analysis, a nomogram was established to predict the probability of NAFLD using age, sex, waist circumference, BMI, ALT and TyG as predictors (Fig. 3). The nomogram is a visualization process of multiple logistic regression analysis. According to the scale corresponding to the top of each predictive variable, the score of the variable could be obtained. The total score is the sum of each single score. When the total score is vertically downward corresponding to Diagnostic possibility, the probability of each individual developing NAFLD could be calculated.

### Validation and calibration of the nomogram

**Figure 4:**
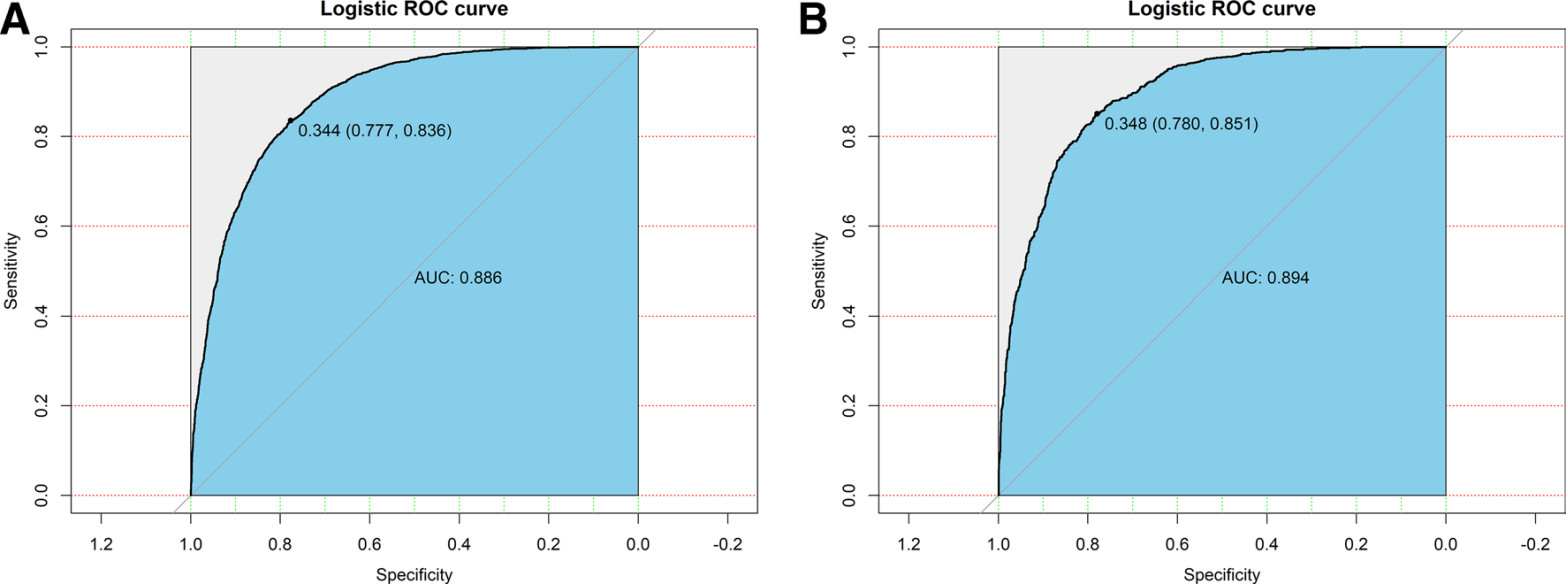
Receiver operating characteristic (ROC) curves validating the discrimination power of the nomogram in the development set (**A**) and the validation set (**B**).

The receiver operating characteristic (ROC) curve was used to evaluate the discriminatory capacity of the nomogram model. The pooled areas under the ROC of the development set and the validation set were 0.886 and 0.894, respectively. The optimal critical value was 0.344 (0.777,0.836) in the development set and 0.348 (0.780,0.851) in the validation set (Fig. 4).

**Figure 5:**
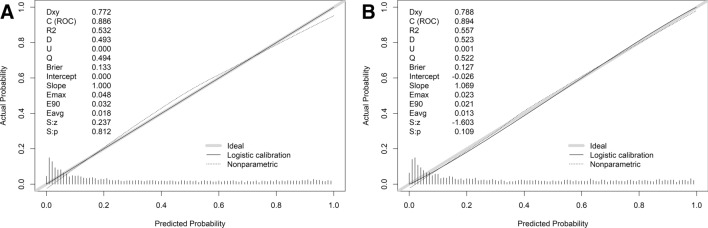
Calibration curves in the development set (**A**) and the validation set (**B**).

A calibration plot and Hosmer–Lemeshow test were applied to calibrate the nomogram model. As shown in Fig. 5, the calibration chart indicated good agreement between the nomogram’s predicted value and the actual observations of NAFLD (development set, *p* = 0.812; validation set, *p* = 0.109). The *P* values in both groups were greater than 0.05, demonstrating the good calibration ability of the model.

## Discussion

The present study found that age, waist circumference, BMI, ALT and TyG were risk factors for the presence of NAFLD, while female sex was a protective factor in a small cohort of NAFLD patients in northwest China.

NAFLD is a disease closely related to insulin resistance and genetic susceptibility that can not only lead to serious liver disease and even death but is also strongly associated with the high incidence of metabolic syndrome, type 2 diabetes and arteriosclerotic cardiovascular disease (ASCVD)^17^. This study observed that the occurrence of NAFLD was relatively parallel to that of obesity in Karamay. In recent years, along with changes in people’s lifestyles, the prevalence of NAFLD has increased rapidly, affecting public health and health-care costs. Therefore, early screening, early diagnosis and timely intervention are of particularly great clinical significance. Since the prevalence of NAFLD varies in different regions, developing a unique and simple screening tool based on conventional screening methods (such as ultrasound) can significantly improve the detection rate of NAFLD. The prediction model is an analytical method that predicts one or more variables based on the correlation between variables. As a quantitative tool of risk and assessment, a clinical predictive model can provide more rational information for doctors, patients and medical policy makers to make decisions; hence, its application is becoming increasingly common^18^. Based on the data from health examinations at Karamay People’s Hospital in Xinjiang, this study constructed a nomogram prediction model of NAFLD to predict the occurrence of NAFLD.

Obesity is a vital risk factor for hypertension, diabetes, dyslipidemia, metabolic syndrome and other diseases. Furthermore, several studies have shown the close relationship between obesity and NAFLD, not only with simple steatosis, but also with NASH, NASH-related cirrhosis and hepatocellular carcinoma^19^. BMI is commonly used to evaluate overweight or obesity. In this study, BMI ≥ 25.0 kg/m^2^ was taken as the overweight/obesity standard. We found that up to 46.97% of the subjects included in this study were overweight or obese. Research has found that despite a lower BMI, Asians may still have visceral fat deposition^20^, which is strongly linked to the risk of cardiovascular disease, diabetes, tumors and other diseases. As a consequence, waist circumference was also included in this study to distinguish abdominal obesity. The number of abdominal obesity cases among all subjects included in the study reached 47.61%. Multivariate logistic regression analysis revealed that both BMI and waist circumference were independent risk factors for NAFLD.

This study also suggested that age and sex played important roles in NAFLD. With the aging of the population, the prevalence rate of NAFLD has increased, the NASH fibrosis score has increased significantly, and the prevalence of liver fibrosis has also increased^21^. Abdominally obese, age-associated visceral fat deposition and secretion of proinflammatory cytokines may be the main reasons^22^. Our study found a higher risk of NAFLD in males, which is similar to previous researches. The underlying mechanism is unclear and may be related to sex hormones and genetic factors^23,24^.

ALT is a specific indicator for liver injury, including hepatic steatosis and steatohepatitis. Research indicated that serum ALT was significantly correlated with increasing stages of fibrosis in NAFLD^25^. The prevalence of NAFLD is generally lower in people with normal ALT^23^. This study also found that serum ALT was an independent risk factor for NAFLD.

Disordered glucose metabolism and dyslipidemia have proven to be critical factors in the occurrence and development of NAFLD^26^, and insulin resistance (IR) can significantly increase the risk of NAFLD^26–29^. Although the hyperinsulinemic-euglycemic clamp is considered the gold standard for the measurement of IR, it is impractical for wide use in clinical diagnosis due to its complex, expensive and time-consuming operation. As an early marker of IR, TyG is calculated from fasting plasma glucose and fasting triglyceride by formula and is often used as a surrogate marker of IR^30,31^. Several studies have reported that TyG is linked to the development of metabolic syndrome, type 2 diabetes and cardiovascular diseases^32^. In recent years, numerous studies have suggested a significant correlation between TyG and NAFLD, which may be a novel predictor for the incidence of NAFLD^16,32,33^. Zheng et al. demonstrated that a threshold of TyG ≥ 8.5 was effective enough to identify NAFLD individuals in a large Chinese population^32^. In a cross-sectional study in Korean adults, the results indicated that TyG was associated not only with the prevalence of NAFLD but also with the severity of NAFLD^33^. Logistic regression analysis of this study also showed that TyG was an independent risk factor for NAFLD, which implied that the occurrence of NAFLD could be prevented by controlling blood glucose, lowering lipids, losing weight and improving insulin resistance.

The variables included in the nomogram of this study are easy to obtain, including age, sex, waist circumference and BMI. Meanwhile, biochemical variables, such as ALT, FPG and TG (to calculate the TyG), are all routinely collected items in medical institutions. In addition, considering the regional differences in the prevalence of NAFLD, this is the first prediction model of NAFLD conducted in Xinjiang on the northwest border of China. According to our research findings, the prevalence of NAFLD in Karamay was as high as 36.8%, which is of great significance for disease prevention in Xinjiang.

There are several limitations of this study. First, this study is a retrospective, single-center, cross-sectional study, which has certain selection bias. Second, some potential risk factors, such as diet and smoking history, were not included in the prediction model. Meanwhile, these factors can also directly affect the TyG value. Third, ultrasound served as the diagnostic reference standard for NAFLD in this study and may be less accurate because the ultrasonic manifestations of diffuse hepatic steatosis and diffuse hepatic fibrosis are similar, which may sometimes be difficult to distinguish. On the other hand, ultrasound cannot accurately quantify the liver fat content, namely, mild, moderate and severe hepatic steatosis. Consequently, more multicenter, long-term follow-up studies are needed for further external validation in the future.

## Conclusions

Predictive models are currently being used in the study of chronic diseases, such as NAFLD, diabetes, hypertension, cardiovascular diseases and other chronic diseases that are endangering public health. Identifying the risk factors for these chronic diseases and establishing predictive models are helpful to reduce the occurrence of these diseases as well as their complications. Through the analysis of clinical and laboratory indicators, this study constructed an NAFLD nomogram prediction model, which is convenient for medical workers to directly analyze the risk of NAFLD. This prediction model can also be used as a supplement to traditional detection to improve the detection rate. Individuals with a high risk of NAFLD could also start early lifestyle interventions and receive health guidance to prevent disease progression and improve their quality of life.

## Data Availability

The data for the current study used for statistical analysis are available from the corresponding author upon reasonable justification.
